# Case Report: Localized bullous pemphigoid induced by local triggers: a case series and a proposal for diagnostic criteria based on a literature review

**DOI:** 10.3389/fimmu.2023.1160779

**Published:** 2023-06-02

**Authors:** Lluís Corbella-Bagot, Javier Gil-Lianes, Javier Fernández-Vela, Ignasi Martí-Martí, Marta Alegre-Fernández, Irene Fuertes, Patricia Garbayo-Salmons, Xavier Bosch-Amate, Antonio Guilabert, José M. Mascaró

**Affiliations:** ^1^ Department of Dermatology, Hospital Clínic de Barcelona, University of Barcelona, Barcelona, Spain; ^2^ Department of Dermatology, Hospital General de Granollers, Granollers, Spain; ^3^ Department of Dermatology, Hospital Mútua de Terrassa, Terrassa, Spain

**Keywords:** bullous pemphigoid, localized bullous pemphigoid, criteria, precipitating factors, triggers, case report, radiotherapy, rosacea

## Abstract

**Introduction:**

Localized bullous pemphigoid (LBP) is an infrequent bullous pemphigoid (BP) variant restricted to a body region. According to the most compelling evidence, LBP occurs in patients with pre-existent serum antibodies against the basement membrane zone, which occasionally acquire the capacity to induce disease after the influence of different local factors acting as triggers.

**Methods:**

We hereby present a multicenter cohort of 7 patients with LBP developed after local triggers: radiotherapy, thermal burns, surgery, rosacea, edema and a paretic leg. In addition, we conducted a review of the literature, and we propose a set of diagnostic criteria for LBP, also based on our case series and the 2022 BP guidelines from the European Academy of Dermatology and Venereology.

**Results:**

During follow-up, three of the patients from our series evolved to a generalized BP, with only one requiring hospitalization. Our literature search retrieved 47 articles including a total of 108 patients with LBP, with a 63% with a potential local precipitating factor previous to their diagnosis. LBP mostly affected older females, and a subsequent generalized progression occurred in 16.7% of the cases. The most frequently involved areas were the lower limbs. Radiation therapy and surgery were responsible for the inducement of nearly 2 in 3 cases of LBP. We observed a significantly higher risk of generalization in cases where the trigger led to the developing of LBP earlier (p=0.016). Our statistical analysis did not detect any other prognosis factor for generalization when assessing direct immunofluorescence, histological and serological results, or other patient related factors.

**Conclusion:**

LBP should be suspected in patients with recurrent localized bullous eruptions. The presence of a trauma history in the same anatomic area is reported in most cases.

## Introduction

Bullous pemphigoid (BP) is the most frequent autoimmune bullous disease, caused by autoantibodies targeting the hemidesmosomal proteins BP180 and/or BP230. It mostly affects the elderly, patients with neurologic conditions, and users of certain medications such as gliptins and anti-programmed cell death protein 1 (anti-PD1) antibodies (1).

BP usually presents with a generalized bullous eruption. In fewer cases (2.5%-29%) lesions appear confined to a particular area: the so-called localized bullous pemphigoid (LBP) (2–4). In 1990, Domloge-Hultsch et al. corroborated that LBP shared the same 230-kd antigen and was a BP subtype (5). The pathophysiology of LBP is not fully understood. Sometimes LBP affects an area with a previous trauma or damage, which would induce the disease. Radiation therapy, surgeries, local burns, ultraviolet radiation and photodynamic therapy are some reported triggers. Latency can range from a few days to several years (6). LBP prognosis is highly varied, from complete resolution to generalization of the lesions.

LBP is however an ill-defined term. Some authors include cases affecting up to 3 body areas (7), while others consider that lesions need to be confined to a single area (8).

Clinical, pathological, serological and immunofluorescence findings resemble those of generalized BP forms. However, there is a lack of standardization regarding its diagnosis criteria. Diagnosis is often delayed as it can mimic other localized bullous diseases. LBP tends to have lower mortality rates compared to generalized BP, and often responds to topical corticosteroids (9). Systemic steroids and immunosuppressants are generally reserved as second-line therapies.

We hereby report 7 cases of LBP, all of them presenting with potential local triggers. We also performed a review of the literature and propose diagnostic criteria for LBP, based on the 2022 BP guidelines from the European Academy of Dermatology and Venereology.

## Materials and methods

A case description of a multicenter cohort and a review of the literature were carried out. We performed a literature search with Pubmed from January-1976 to December-2022 using the keywords “localized” and “bullous pemphigoid”. The search strategy was restricted to English language articles. Reports and reviews describing localized forms of bullous pemphigoid were analyzed. We included LBP patients with lesions restricted to a single cutaneous area. We also included cases of bilateral involvement in limbs, and cases with two contiguous body areas involved at the site of a previous trigger. Importantly, generalized BP occasionally presents with initially localized lesions. In line with the proposal from Ständer et al., we excluded cases where generalization had been reported prior to 3 months after the onset of the localized disease (4). We also excluded cases of dyshidrosiform BP, localized mucosal lesions, localized cicatricial pemphigoid (Brunsting-Perry), localized forms in patients with previous generalized BP, cases with insufficient data or without a closed diagnosis, and cases described as “localized” but with several anatomical areas involved.

Statistical analyses were performed using the Statistical Package for the Social Sciences (SPSS). Quantitative variables were summarized with means or medians and standard deviations or interquartile ranges. Categoric variables were reported as percentages. Comparisons between groups to identify predictor variables for generalized BP were performed using a Chi-squared or a Fisher’s exact test. P values < 0.05 were considered statistically significant.

## Results

Our series includes 7 patients with a mean age of 72.3 (± 9.1) years and a male predominance (71.4%). LBP was attributed to radiation therapy in 28.6% of the cases. Mean latency from the trigger was 12.8 (± 18.3) months. Chest (42.9%) and lower limbs (28.6%) were the most frequent locations. Subsequent generalization of BP took place in 42.9% of the patients. In all cases in which histology and immunofluorescence were performed, positive results were observed, while anti-BP180 and anti-BP230 had a positivity rate of 75% and 25% among patients tested, respectively (**Table 1**)

**Table 1:** Cohort of localized bullous pemphigoid patients from the present study.

N.	Age, yrs.	Sex	Local trigger	Latency	Other contributing factors	Affected Area	Treatment	Generalization of BP (time elapsed from LBP onset)	Histology	ELISA	DIF	IIF
1	72	F	Radiation therapy	1 week after last RT session	Sitagliptin	Right chest	Topical and systemic corticoids. Topical antibiotics	Yes (10 months)	Subepidermal blister with abundant eosinophils.	Anti-BP180:+ Anti-BP230:+	Linear C3 deposits along the BMZ.	Circulating IgG autoantibodies against the BMZ on monkey esophagus, which also bound the epidermal side of 1M salt-split-skin BMZ.
2	87	M	Thermal burns	1-2 months	None	Abdomen	Topical corticoids	No	Subepidermal blister with a dermal infiltrate composed of eosinophils and lymphocytes.	Not performed	Linear IgG and C3 deposits along the BMZ.	Not performed
3	76	M	Vascular access device implantation.	3-4 weeks	Anti-PD1 drug.	Right chest	Topical antibiotics and corticoids	No	Subepidermal blister with presence of eosinophils.	Not performed	Linear IgG, C3 and mild IgM deposits along the BMZ.	Not performed
4	57	M	Radiation therapy	49 months	Anti-PD1 drug.	Right chest	Topical corticoids (initially)	Yes (14 months)	Subepidermal blister with presence of eosinophils.	Anti-BP180:+ Anti-BP230:-	Linear IgG, C3 and mild IgA deposits along the BMZ with an n-serrated pattern.	Positive, circulating IgG antibodies against the epidermal side of the BMZ.
5	68	F	Orthopedic surgery	1 month	Parkinson’s disease	Lower left limb	Doxycycline, oral prednisone, methotrexate	Yes (3 months)	Suggestive of BP.	Anti-BP180:+ Anti-BP230:-	Linear deposits of IgG and C3 along the BMZ with areas of subepidermal vesiculation. IgG deposits on the epidermal side of the blister.	Not performed
6	78	M	Rosacea	NA	None	Nose	Topical corticoids	No	Subepidermal blister with a predominance of dermal eosinophils.	Anti-BP180:- Anti-BP230:-	Linear deposits of IgA (+), IgG (++), and C3 (+++), with a higher intensity on the epidermal side of the BMZ.	Not performed
7	82	M	Paretic leg	2 years	None	Lower right limb	Topical corticoids	No	Subepidermal blister with a predominance of eosinophils.	Not performed	Linear deposits of IgG and C3 along the BMZ.	Not performed

N., Case number; BP, Bullous pemphigoid; M, Male; F, Female; RT, Radiotherapy; BMZ, Basement membrane zone; DIF, Direct immunofluorescence; IIF, Indirect immunofluorescence; LBP, Localized Bullous Pemphigoid; NA, Not Available.

### Case 1

**Figure 1:**
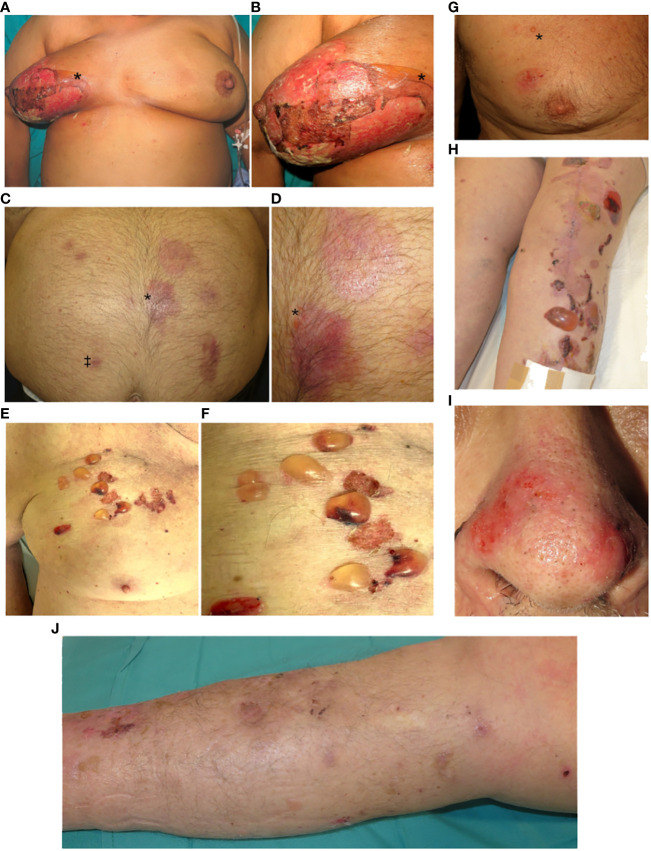
Localized bullous pemphigoid (LBP). **(A, B)** Case 1. Radiation-induced LBP: Multiple skin erosions confined to the right breast. On the upper medial quadrant a flaccid blister can be distinguished (*). **(C, D)** Case 2. Thermal burns-induced LBP: Multiple plaques in the abdomen 6 months after an accidental burn. Note the presence of milia cysts (‡) and a small tense vesicle (*). **(E, F)** Case 3. LBP induced by Central venous port insertion: Serous and sero-hemorrhagic tense blisters confined to the upper right chest. Note a small protuberance near the scar corresponding to the Port-a-Cath^®^ device. **(G)** Case 4. Radiation-induced LBP: Presence of erosions and a tense vesicle localized on the right chest (*). **(H)** Case 5. LBP induced by orthopedic surgery: Tense bullae, erosions and crusting over an erythematous base located predominantly over the surgical scar. **(I)** Case 6. LBP induced by rosacea: Multiple erosions over an erythematous base on the nasal tip. **(J)** Case 7. LBP induced by right hemiparesis: Presence of erosions, crusting and post-inflammatory pigmentation on the right lower limb.

**Figure 2:**
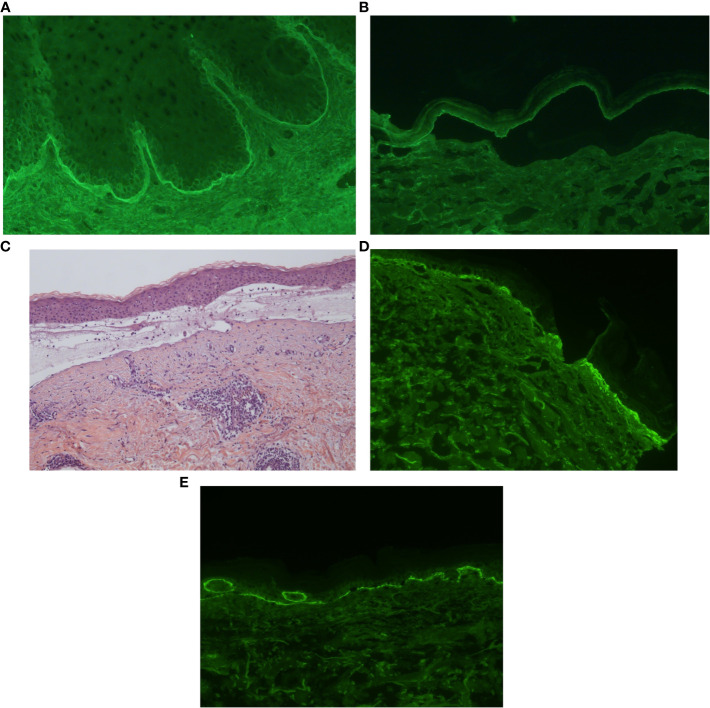
Histology and immunofluorescence studies of localized bullous pemphigoid (LBP). **(A, B)** Indirect immunofluorescence of radiation-induced LBP: IgG autoantibodies bound to the basement membrane zone (BMZ) of monkey esophagus **(A)**. These IgG autoantibodies also bound to the epidermal side of 1M salt-split-skin BMZ **(B)**. **(C–E)** Histology and direct immunofluorescence (DIF) of LBP induced by thermal burns. Images show a subepidermal blister with superficial dermal mixed inflammatory infiltrate composed of eosinophils and lymphocytes **(C)**. Direct immunofluorescence showed linear IgG **(D)** and C3 **(E)** deposits on the BMZ.

A 72-year-old woman, with a history of diabetes managed with sitagliptin/metformin, was treated with tumorectomy and adjuvant radiotherapy for a breast cancer. One week after radiotherapy, she presented painful erosions, crusts, and a large bulla confined to the irradiated breast (**Figures 1A, B**). Histology revealed a subepidermal blister with abundant eosinophils. Direct immunofluorescence examination (DIF) showed linear deposition of C3 along the basement membrane zone (BMZ). Indirect immunofluorescence examination (IIF) showed the presence of circulating IgG autoantibodies against the BMZ, which bound the epidermal side of 1M salt-split-skin BMZ (**Figures 2A, B**). ELISA studies detected autoantibodies against both BP180 (234 U/mL, normal values <20 U/mL) and BP230 (70 U/mL, normal values <20 U/mL). Sitagliptin was withdrawn and systemic and topical corticoids were administered. Three months after the onset of the bullous eruption, the lesions epithelized, and oral corticosteroids could be successfully withdrawn. The patient remained free of oral corticosteroids and with clinical stability for a total of 4 consecutive months, until a new localized flare in the same area required their restart. Few days after this second course of corticosteroids was progressively tapered to 10 mg daily, the patient developed a generalization of her BP that required hospitalization. At follow up, she has presented new flares mainly localized to her right breast.

### Case 2

An 87-year-old male with a history of cardiopathy suffered an accidental scalding in the abdomen. One month later, he started with asymptomatic recurrent vesicles on the previously wounded area. Physical exam revealed multiple erythematous to violaceous plaques, milium cysts and a vesicle on the edge of a plaque (**Figures 1C, D**). Histology showed a subepidermal blister with a mixed lympho-eosinophilic infiltrate (**Figure 2C**). DIF revealed lineal deposition of IgG and C3 along the BMZ (**Figures 2D, E**). IIF and ELISA assays were not performed. Topical corticosteroids were started with a complete remission at follow-up.

### Case 3

A 76-year-old male had a history of metastatic lung adenocarcinoma. A vascular access device with a reservoir (Port-a-Cath®) was implanted for the administration of pembrolizumab. A few days later, an erythematous plaque with vesicular lesions appeared restricted to the skin under the adhesive tape. Contact dermatitis triggered by adhesive tape was suspected, and presented remission when the tape was removed. However, after 3 weeks the patient started presenting serohemorrhagic tense bullae and several erosions on the same area (**Figures 1E, F**). Histology showed a subepidermal blister with presence of eosinophils, and DIF showed linear IgG, C3 and mild IgM deposition along the BMZ. IIF and ELISA assays were not performed. Topical corticosteroids were prescribed with a complete remission. Immunotherapy was not interrupted. No relapses have been detected after 6 months of follow-up.

### Case 4

A 57-year-old male had a history of lung adenocarcinoma treated with surgery, chemotherapy, and radiation therapy. Twenty months later, nivolumab was initiated due to metastatic progression. After 29 months, the patient presented with a tense vesicle, multiple erosions and scars on the irradiated right pectoral area (**Figure 1G**). A subepidermal vesicle with eosinophils was observed in the biopsy, and linear IgG, C3 and IgA deposits along the BMZ were shown on DIF. ELISA studies detected autoantibodies against BP180 (74 U/mL, normal values <20 U/mL). IIF showed circulating linear IgG deposits against the epidermal side of the BMZ. Topical corticosteroids were started, with a complete remission. On follow-up, nivolumab was interrupted and oral prednisone was started due to an immune-mediated pneumonitis. A generalized BP flare occurred during the corticoids tapering, which required treatment with prednisone 0.5 mg/kg/day, with no further relapses after 3 years.

### Case 5

A 66-year-old woman with Parkinson’s disease underwent knee replacement surgery. One month later, she developed bullous lesions on the operated leg, predominantly over the surgical scar (**Figure 1H**). Histology was compatible with BP. DIF showed linear deposits of IgG and C3 along the BMZ, with IgG deposits located in the epidermal side of the blister. IIF was not performed. ELISA study showed BP180 autoantibodies (79 U/mL, normal values <20 U/mL). Doxycycline with gradually tapered systemic corticosteroids were administered with a good initial response. Three months after the eruption, the patient presented scattered lesions in the arms that responded to topical corticosteroids and a short course of methotrexate. The patient did not present new lesions over one year of follow-up.

### Case 6

A 78-year-old man with papulopustular rosacea with nasal involvement presented with a 2-year history of vesicular lesions with erosions and crusting on the nasal tip (**Figure 1I**). Histology showed a subepidermal blister with abundant dermal eosinophils. DIF revealed linear deposits of IgA (+), IgG (++), and C3 (+++) along the BMZ with a higher intensity in the epidermal side of the blister. IIF was not performed. ELISA exam was negative. The patient presented a complete response to topical corticosteroids.

### Case 7

A 77-year-old male with a history of ischemic stroke with residual right hemiparesis, presented two years later with pruritic papules and blisters restricted to the hemiparetic right lower extremity (**Figure 1J**). Histology showed a subepidermal blister with a mixed infiltrate composed predominantly of eosinophils. DIF revealed linear deposits of IgG and C3 along the BMZ. Serologic studies were not performed. Topical corticosteroids were started with a good response without relapse over the next 6 months.

### Cases from a literature review

Of a total of 239 reviewed articles, we selected 47 articles with 101 patients matching the inclusion criteria. With our case series, 108 patients were included (**Supplementary Table 1**).

The mean age at diagnosis was 71.2 (± 14.4) years, with a female predominance (66.7%). A local trigger was identified in 63% of the cases, being radiation therapy (32.3%) and surgery (29.4%) the most frequently reported. Median latency was 150 days. Other contributing factors were reported in 26.9% of the cases. The most frequently affected areas were the lower limbs (43.9%). Topical corticosteroids were administered as the exclusive treatment in 46.6% of the cases. Generalization of BP was reported in 16.7% of the patients, after a median of 5 months from the onset of symptoms. Histological analysis yielded positive results in 86.8% of the cases, defined as a subepidermal blister with eosinophils, or alternatively a description of a “positive” or “compatible” biopsy. DIF and IIF reported positive results in 94.4% and 85.1% of the cases, respectively. Other serological analysis testing for the presence of anti-BP180 and anti-BP230 antibodies were reported in 38.9% of the cases, with a positivity of 76.2% (anti-BP180 48.7%, anti-BP230 41.0%) (**Table 2**). Statistical analysis detected a significant relationship between the latency time and the risk of generalization (p=0.016), showing a higher risk in cases in which the local factor quickly triggered the disease. No association was observed between the risk of progression and age, sex, affected area, presence or type of local trigger, compatible histology; or positive DIF, IIF, BP180 and/or BP230 antibodies (**Table 3**).

**Table 2:** Literature review – clinical presentation, diagnosis, treatment and prognosis of localized bullous pemphigoid retrieved from Pubmed search engine and our case series (n=108).

	N (%)
Sex	
Male	70 (33.3%)
Female	35 (66.7%)
Age	
Mean years (± SD)	71.2 (± 14.4)
Local trigger	
**Yes**	68 (63.0%)
Radiation therapy	22 (32.3%)
Surgery	20 (29.4%)
Stoma	6 (30%)
Hemodialysis fistula	2 (10%)
UV exposure	4 (5.9%)
Phototherapy	1 (25%)
Infection	3 (4.4%)
Edema/lymphedema	2 (2.9%)
Thermal burns	2 (2.9%)
Injury	2 (2.9%)
Other	13 (19.1%)
**No**	40 (37.0%)
Latency	
Median months (IQR)	5 (47.9)
Affected area	
Lower limb(s)	47 (43.9%)
Chest	21 (19.7%)
Chest + other contiguous areas	2 (10.5%)
Upper limb(s)	14 (13.1%)
Abdomen	9 (8.4%)
Genital	6 (5.6%)
Facial	4 (3.7%)
Axillar	2 (1.8%)
Other	4 (3.8%)
Treatment	
Topical corticosteroids in monotherapy	34 (46.6%)
Oral corticosteroids in monotherapy	7 (9.6%)
Combination of topical and oral corticosteroids	10 (13.7%)
Combination of topical, intralesional and topical corticosteroids	2 (2.7%)
Oral corticosteroids and topical tacrolimus	2 (2.7%)
Tetracyclines-based regimens	5 (6.8%)
Niacinamide-based regimens	1 (1.4%)
Niacinamide and tetracyclines in combination	2 (2.7%)
Others	10 (13.7%)
Subsequent generalization	
Yes	18 (16.7%)
No/Not referred	90 (83.3%)
Time elapsed from LBP onset to BP diffuse progression	
Median months (IQR)	5 (6)
Histology	
Performed	68 (63.0%)
Subepidermal blister with eosinophils/Positive (description unavailable)	59 (86.8%)
Negative/Other findings	9 (13.2%)
Not performed/not mentioned	40 (37.0%)
Serological presence of anti-BP180 and anti-BP230 antibodies	
Performed	42 (38.9%)
Anti-BP180 positivity only	15 (35.7%)
Anti-BP230 positivity only	12 (28.6%)
Anti-BP180 and anti-BP230 positivity	4 (9.5%)
Positive (not specified)	1 (2.4%)
Negative	10 (23.8%)
Not performed/not mentioned	66 (61.1%)
DIF	
Performed	90 (83.3%)
Positive	85 (94.4%)
Negative	5 (5.6%)
Not performed/not mentioned	18 (16.7%)
IIF	
Performed	67 (62.0%)
Positive	57 (85.1%)
Negative	10 (14.9%)
Not performed/not mentioned	41 (38.0%)

BP, Bullous pemphigoid; LBP, Localized bullous pemphigoid; SD, standard deviation; IQR, interquartile range.

**Table 3:** Literature review – Statistical analysis by means of a Chi-squared test to assess potential associations to the risk of subsequent generalization in the reported sample (n=108).

Variable	No/non referred subsequent generalization	With subsequent generalization	Difference (%)	p-value (CI 95%)
Sex			-13.4%	0.272 (-0.0827, 0.351)
Female	56 (64.4%)	14 (77.8%)		
Male	31 (35.6%)	4 (22.2%)		
Age (years)			-6.7%	0.411 (-0.250, 0.116)
≤50	9 (10.0%)	3 (16.7%)		
>50	81 (90.0%)	15 (83.3%)		
Local trigger			-17.8%	0.154 (-0.395, 0.0393)
Yes	54 (60.0%)	14 (77.8%)		
No	36 (40.0%)	4 (22.2%)		
Local trigger				0.294
RT	17 (31.5%)	5 (35.7%)	-4.2%	
Surgery	14 (25.9%)	6 (42.9%)	-27.0%	
Other	23 (42.6%)	3 (21.4%)	21.2%	
Latency (days)				0.016
≤15	4 (11.8%)	6 (50.0%)	-38.2%	
16-60	9 (26.5%)	3 (25.0%)	1.5%	
>60	21 (61.8%)	3 (25.0%)	36.8%	
Affected area				0.215*
Chest (exclusively)	16 (18.0%)	3 (16.7%)	1.3%	
Abdomen	8 (9.0%)	1 (5.6%)	3.4%	
Axilla	2 (2.2%)	0 (0%)	2.2%	
Upper limb	12 (13.5%)	2 (11.1%)	2.4%	
Lower limb	39 (43.8%)	8 (44.4%)	-0.6%	
Site of irradiation	1 (1.1%)	0 (0%)	1.1%	
Genital	6 (6.7%)	0 (0%)	6.7%	
Chest and other contiguous areas	2 (2.2%)	0 (0%)	2.2%	
Inguinal	0 (0%)	1 (5.6%)	-5.6%	
Facial	3 (3.4%)	1 (5.6%)	-2.2%	
Back	0 (0%)	1 (5.6%)	-5.6%	
Neck	0 (0%)	1 (5.6%)	-5.6%	
Treatment				0.723*
Topical corticosteroids	27 (45.8%)	7 (50.0%)	-4.2%	
Oral corticosteroids	5 (8.5%)	2 (14.3%)	-5.8%	
Topical and oral corticosteroids	6 (10.2%)	4 (28.6%)	-18.4%	
Niacinamide and tetracycline	2 (3.4%)	0 (0%)	3.4%	
Topical, systemic and IL corticoids	2 (3.4%)	0 (0%)	3.4%	
Oral corticoids and topical tacrolimus	2 (3.4%)	0 (0%)	3.4%	
Other regimens Other niacinamide-based regimens	9 (15.3%) 1 (1.7%)	1 (7.1%) 0 (0%)	8.2% 1.7%	
Other tetracycline-based regimens	5 (8.5%)	0 (0%)	8.5%	
Histology			-6.0%	0.581 (-0.122, 0.241)
Positive	48 (85.7%)	11 (91.7%)		
Negative	8 (14.3%)	1 (8.3%)		
Anti-BP180 and anti-BP230 antibodies			-9.8%	0.609 (-0.253, 0.431)
Positive	25 (73.5%)	5 (83.3%)		
Negative	10 (26.5%)	1 (16.7%)		
Direct immunofluorescence			-6.2%	0.340 (0.00932, 0.114)
Positive	76 (93.8%)	14 (100%)		
Negative	5 (6.2%)	0 (0%)		
Indirect immunofluorescence			0.9%	0.939 (-0.226, 0.209)
Positive	47 (85.5%)	11 (84.6%)		
Negative	8 (14.5%)	2 (15.4%)		

BP, Bullous pemphigoid; LBP, Localized bullous pemphigoid; IL, Intralesional; SD, standard deviation; IQR, interquartile range; CI, Confidence Intervale; RT, Radiotherapy. * Calculation performed by means of Fisher’s exact test.

## Discussion

LBP is a variant of BP confined to a single body site, with similar clinical and immunopathologic features to generalized forms, yet with some singularities: a high prevalence of localized triggers, a better prognosis (9), higher chances of therapeutic success with topical corticosteroids, and a risk of generalization.

All cases from our series involved potential triggers. In Case 1 the patient was under gliptins, a pharmacological trigger for BP. She developed LBP one week after completing radiotherapy in her right breast. In this presentation, generalized pruritus and bulla formation were decisive to differentiate BP from radiodermatitis. Oral corticosteroids were required since the affected breast was almost completely denudated and topical treatment would have most likely been insufficient. In our review, radiotherapy was the most frequent trigger reported in LBP, being breast cancer the most frequent neoplasm. In 69% of the cases, LBP appears within the first 6 months after radiotherapy and in 38%, irradiated LBP evolved to generalized BP (10), as it occurred in Cases 1 and 4. It has been observed that radiation enhances 2 to 3-fold autoantibody binding to the BMZ (11). Radiotherapy induces tumor and epidermal cell death, but Langerhans cells have shown resistance to radiation-induced apoptosis. Hence, these cells would present the exposed antigens to CD4+ T cells, eventually leading to autoantibody formation, complement activation and proinflammatory cytokine secretion. Breast cancer cells have shown to express hemidesmosomes *in vitro*. Thus, radiotherapy-induced apoptosis could release BP180 and BP230 antigens, which may become immunogenic and lead to autoantibody production (10).

Case 2 was triggered by thermal burns, which have been reported as rare inductors of BP and pemphigus vulgaris (12). Cases 3 and 5 were triggered by surgeries, one of the most frequent triggers of LBP, although we have not found any report of LBP triggered by Port-a-Cath^®^ implant surgery in the literature. We believe that in Case 3 the prior diagnosis of contact dermatitis was in fact an early manifestation of LBP. Due to their clinical similarities, early manifestations of LBP have been misdiagnosed as contact dermatitis in several cases (13), which is an infrequent but also reported cause of LBP (14). Pembrolizumab (Case 3) and nivolumab (Case 4) are anti-PD1 monoclonal antibodies with the potential to induce LBP. However, both patients did not develop new lesions with subsequent drug administrations. Even though some inflammatory cutaneous diseases have been described as triggers for LBP (13, 15, 16), Case 6 is to our knowledge the first reported case secondary to rosacea. In rosacea, DIF may be not specific of LBP, since an IgG, IgM or C3 band, with or without dermal deposits, have been observed in 39% of the cases (17) and histological features of BP would be mandatory for the diagnosis. In Case 7, skin lesions were confined to the hemiparetic limb, a rare but reported presentation of LBP (18–22). Association between neurologic disorders and BP is well known. It has been proposed that local neuromuscular and vascular changes in the paretic leg, together with immobility and scratching can contribute to the onset of LBP lesions (19). Moreover, an injured cutaneous site, such as the lymphedematous paretic limb, may become an immunocompromised district in which the neuro-immunocutaneous system would become destabilized, predisposing the area to the development of secondary diseases, including autoimmune disorders (22). This mechanism has been proposed to act more intensely on late-onset induced LBPs (23). Differential diagnosis of LBP includes *bullosis diabeticorum*, bullous drug eruption, insect bite reaction, other autoimmune bullous diseases and viral infections. Potent topical corticosteroids usually suffice for disease control. For extended or refractory cases, systemic corticosteroids and/or other immunosuppressants can be considered.

The mechanisms underlying how LBP is triggered are not fully understood. It has been demonstrated with mouse models with epidermolysis bullosa acquisita how skin areas exposed to mechanical irritation showed increased autoantibody binding along the dermoepidermal junction and severe clinical manifestations (24).

According to one hypothesis evaluated ex-vivo, LBP would present in initially asymptomatic, genetically susceptible individuals with pre-existent serum antibodies against the BMZ. These autoantibodies would bind to BMZ proteins without inducing the disease. After tissue destruction, the wound remodeling process would stimulate the synthesis of vascular endothelial growth factor (VEGF), increasing vascular permeability, leading the migration of granulocytes and other inflammatory cells, and enhancing anti-BP180 and anti-BP230 circulation. The presence of granulocytes in the BMZ would trigger the disease by binding to these autoantibodies, activating the complement system (6).

The results from our literature review show a clear female preponderance in LBP with twice as many cases in females than in males, which is consistent with a female-to-male ratio of 1.04-5.1 reported in BP (25). Cases triggered by radiation therapy in breast cancer and the female predominance among the elderly population can explain these results. Radiation therapy and surgery were the most frequent triggers in LBP, though these patients can be overrepresented considering that some case series included were restricted to patients with these triggers. Interestingly, we found that 16.7% of the cases presented generalization of lesions. The most frequently affected areas were the lower limbs. In line with Kohroh et al. (26), we believe that increased hydrostatic pressure, friction due to socks and pretibial microtrauma can play a role in LBP development in these regions. Latency from the local trigger to the onset of the bullous eruption ranged from 2 days to 47 years. Even though lower rates of positive results in DIF and in anti-BP180 ELISA compared to generalized BP had been detected in previous studies (8, 27), we found a 94.4% positivity in DIF and 76.2% in ELISA studies. Interestingly, a study showed an increased auto-reactivity in LBP to BP230 IgG compared to classic BP (28). We only found higher risk for generalization in cases in which the local trigger induced the disease within the first 15 days. On the other hand, cases induced by a trigger with more than 60 days of latency had a lower risk for generalization. As mentioned previously, it has been proposed that LBP could be induced by different mechanisms in early and late-onset triggered LBP. Thus, the generalization risk could differ too. Further research is needed to elucidate the implications of this association. The lack of differences in test results compared to cases that remain localized support the theory that LBP diagnosis should not be retrospectively discarded in cases which get generalized after at least 3 months. Thus, we believe that both these subtypes correspond to the same variant of BP.

Limitations of this study include the variable follow-up time and the designation of triggers with a temporal and spatial relationship with LBP onset which may not always be responsible for LBP. In articles where the performance of serological, histological or DIF exams was not mentioned, we could not discern reliably between negative and not performed results. The prerequisite of 3 months of localized activity to accept the diagnosis and discard an early classic form of BP is arbitrary, and could require adjustments in further studies as more knowledge on the field is available. Due to the retrospective nature of the study, results shown can also be influenced by a publication bias.

## Diagnostic criteria for Localized Bullous Pemphigoid

Diagnosis of LBP is often delayed, and misdiagnosis is not rare (29). With the purpose to contribute to early diagnosis, we propose that LBP diagnosis could be based on an adaptation of the recently published criteria for BP (30). LBP will be confirmed by the fulfillment of three of the following four criteria, with the clinical criteria being mandatory:

1. **Clinical criteria**:

Presence of a bullous eruption confined to a single anatomic region, sometimes preceded by a predisposing factor (“triggered LBP”), without a previous history of generalized BP. In the event of a subsequent generalization, lesions must have remained localized for at least 3 months and patients should not have received systemic corticosteroids in the intervening period. *

2. **DIF criteria**:

Positive DIF with linear deposits of IgG and/or C3 along the BMZ (preferably with an n-serrated pattern). Sometimes IgA and IgE with a similar pattern.

3. **Serological criteria**:

Positive IgG antibodies against the epidermal side of BMZ by IIF.

and/or

Positive IgG antibodies reacting with BP180 and/or BP230 by ELISA, IIF, immunoblot, or immunoprecipitation.

4. **Histological criteria**:

Subepidermal blister with the presence of eosinophils.

*In localized lesions with absence of blisters, LBP can be accepted in patients fulfilling both DIF and serological criteria.

## Conclusion

LBP should be considered in patients presenting recurrent local bullous eruptions, especially in patients with a history of exposure to any known trigger, which are responsible for 63% of LBP. Diagnostic confirmation using DIF, serology and histology is required as in other variants of BP. Even though we have not found any prognosis factor for generalization in LBP among complementary tests, we believe that serologic exams and histology are crucial for a reliable diagnosis. We propose new diagnostic criteria for LBP to aid rapid diagnosis and direct treatment. Topical corticosteroids are enough for the control of milder forms of LBP, but at least half of the patients will require systemic therapies at some point of their disease.

## Data availability statement

The original contributions presented in the study are included in the article/**Supplementary Material**. Further inquiries can be directed to the corresponding author.

## Ethics statement

Ethical review and approval was not required for the study on human participants in accordance with the local legislation and institutional requirements. The patients/participants provided their written informed consent to participate in this study. Written informed consent was obtained from the individual(s) for the publication of any potentially identifiable images or data included in this article.

## Author contributions

LC-B wrote the first draft, performed the review of the literature and statistical analysis, and wrote the final version. JM contributed to conception of the work, and leaded in the writing. JG-L contributed in the design of the study and statistical analysis and performed multiple reviews of the text. JF-V wrote sections of the manuscript. XB-A contributed in the design of the study and performed multiple reviews of the text. AG leaded in the description of the patients from Hospital de Granollers and in the design of the study. All authors (LC-B, JM, JG-L, JF-V, XB-A, AG, PG-S, IM-M, MA-F, IF) participated in the inclusion of patients and their data submission to our case series. All authors contributed to the article and approved the submitted version.
