# Supplementary material for: Case Report: Localized bullous pemphigoid induced by local triggers: a case series and a proposal for diagnostic criteria based on a literature review
Source: Front Immunol. 2023 Jun 2;14:1160779. doi: 10.3389/fimmu.2023.1160779 (PMC10272756; doi:10.3389/fimmu.2023.1160779)
Supplement: Supplementary file 1 [file Table_1.docx]

**Supplementary Tables**

**Supplementary Table 1.** Cohort of Localized bullous pemphigoid patients (n=108).

| **Ref.** | **Age** | **Sex** | **Local trigger** | **Latency (days)** | **Other contributing factors** | **Affected Area** | **Treatment** | **Generalization** | **Months to generalization** | **Histology** | **Anti-BP180 and BP230 positivity** | **DIF** | **IIF** |
| --- | --- | --- | --- | --- | --- | --- | --- | --- | --- | --- | --- | --- | --- |
| PS | 72 | F | Radiotherapy | 7 | Sitagliptin | Chest | Topical and systemic corticoids | Yes | 10 | Subepidermal blister with eosinophils. | BP180, BP230 | C3 | IgG, epidermal side |
| PS | 87 | M | Thermal burns | 45 | None | Abdomen | Topical corticoids | No/not referred | - | Subepidermal blister with eosinophils. | Not performed | IgG and C3 | Not performed |
| PS | 76 | M | Vascular access device implantation. | 25 | Anti-PD1 | Chest | Topical corticoids | No/not referred | - | Subepidermal blister with eosinophils. | Not performed | IgG, C3, IgM | Not performed |
| PS | 57 | M | Radiotherapy | 1470 | Anti-PD1 | Chest | Topical corticoids | Yes | 14 | Subepidermal blister with eosinophils. | BP180 | IgG, C3, IgA | IgG, epidermal side |
| PS | 82 | M | Paretic leg | 730 | None | Lower limb | Topical corticoids | No/not referred | - | Subepidermal blister with eosinophils. | Not performed | IgG and C3 | Not performed |
| PS | 68 | F | surgery | 30 | Parkinson's disease | Lower limb | Tetracycline + oral corticoids + MTX | Yes | 3 | Compatible | BP180 | IgG and C3 | Not performed |
| PS | 78 | M | Rosacea | NA | None | Nose | None | No/not referred | - | Subepidermal blister with eosinophils. | Negative | IgA, IgG, C3 | Not performed |
| 1 | 84 | M | stoma | - | - | Abdomen | Topical corticoids | No/not referred | - | - | BP180, BP230 | IgG and C3 | Epidermal side |
| 1 | 68 | M | Radiotherapy | - | - | Axilla | Topical corticoids | No/not referred | - | Subepidermal blister with eosinophils. | Negative | IgG and C3 | Epidermal side |
| 1 | 72 | F | surgery | 60 |  | Upper limb | Topical corticoids + dapsone | Yes | 3 | - | BP180 | - | Epidermal side |
| 1 | 61 | F | Radiotherapy | - | PEG tub insertion | Cervical (neck) | - | Yes | 18 | - | BP180 | C3 | Epidermal side |
| 1 | 62 | F | Lymphedema | - | Mastectomy | Upper limb | Topical corticoids + ethiological | No/not referred | - | - | BP180 | IgG and C3 | Epidermal side |
| 1 | 69 | F | Bacterial infection | - | - | Lower limb | Ethiological | No/not referred | - | - | BP180 | IgG and C3 | Epidermal side |
| 1 | 61 | F | Bacterial infection | 5 | - | Lower limb | Topical corticoids | No/not referred | - | - | BP180 | C3 | Epidermal side |
| 2 | 75 | M | - | - | - | Scrotum | MMF, oral corticoids, doxycycline | No/not referred | - | Spongiosis, ulceration, presence of eosinophils | Negative | IgG and C3 | IgG (monkey esophagus) |
| 3** | 85 | F | surgery | 2 | - | Lower limb | Topical corticoids | Yes | 5 | - | Negative | - | Negative |
| 3 | 74 | M | surgery | 270 | - | Lower limb | Topical corticoids | No/not referred | - | - | - | IgG and C3 (epidermal side) | - |
| 3 | 76 | F | surgery | 14 | - | Feet | Oral corticoids | Yes | - | Compatible | - | Compatible (IF studies) | Compatible (IF studies) |
| 4 | 81 | M | stoma | 2555 | - | Abdomen (peristomal) | Topical corticoids | yes | 4 | Subepidermal blister with eosinophils. | BP180 | IgG and C3 (epidermal side) | IgG, epidermal side |
| 3 | 67 | F | surgery | 18250 | - | Upper limb (Split skin graft) | - | No/not referred | - | - | - | - | - |
| 5 | 66 | M | Surgery | 7300 | Furosemide | Lower limb (Split skin graft) | Topical corticoids | No/not referred | - | Subepidermal blister with eosinophils. | - | IgG and C3 | Negative |
| 3 | 76 | M | surgery | 2555 | - | Upper limbs | Oral corticoids | No/not referred | - | Necrosis with eosinophils | Negative | IgG, C3, IgM | - |
| 3 | 52 | F | surgery | 3285 | Plaque psoriasis | Upper limb | Oral corticoids | No/not referred | - | Compatible | - | Compatible | - |
| 3 | 66 | M | surgery | 60 | - | Lower limb (Stump) | Topical and oral corticoids | No/not referred | - | Subepidermal blister with eosinophils. | Negative | IgG (epidermal side) | - |
| 6 | 55 | F | Radiotherapy | 1080 | Lymphedema | Chest | - | No/not referred | - | Compatible | - | Compatible | - |
| 6 | 57 | F | Radiotherapy | 21 | Surgery | Chest + neck | Topical corticoids | No/not referred | - | Subepidermal blister with eosinophils. | - | IgG and C3 | IgG, epidermal side |
| 7 | 58 | F | Radiotherapy | 150 | Surgery | Chest | Niacinamide | No/not referred | - | Compatible | - | Compatible | Negative |
| 6 | 58 | F | Radiotherapy | 60 | - | Chest | Niacinamide + tetracycline | No/not referred | - | Compatible | - | Compatible | Compatible |
| 8 | 65 | F | Radiotherapy | 150 | Surgery | Chest | Topical corticoids | No/not referred | - | Subepidermal blister with eosinophils. | - | IgA, IgM,C3 | Negative |
| 9 | 66 | F | Radiotherapy | 30 | Surgery | Chest | Tetracycline + oral corticoids | No/not referred | - | Subepidermal blister with eosinophils. | - | IgG and C3 (epidermal side) | Epidermal side |
| 8 | 76 | F | Radiotherapy | 480 | Surgery | Chest | Topical corticoids | No/not referred | - | Subepidermal blister with eosinophils. | BP230 | IgG and C3 | IgG |
| 6 | 77 | F | - | ** | Radiotherapy (outside the field) | Right inguinal region | Topical corticoids | Yes | 3 | - | - | IgG | Compatible |
| 10 | 78 | F | Radiotherapy | 1080 | Surgery | Chest | - | Yes | 7 | Compatible | - | Compatible | Compatible |
| 6 | 78 | F | Radiotherapy | 1440 | - | Chest | - | No/not referred | - | Compatible | - | Compatible | Compatible |
| 6 | 78 | F | Radiotherapy | 365 | - | Chest | - | No/not referred | - | Compatible | - | Compatible | Compatible |
| 6 | 79 | F | Radiotherapy | 150 | - | Chest | - | No/not referred | - | Subepidermal blister with eosinophils. | - | IgG and C3 | IgG, IgM, C3 |
| 6 | 80 | F | Radiotherapy | ** | Surgery | Chest | Topical corticoids | No/not referred | - | Not performed | Not performed | Not performed | Not performed |
| 6 | 81 | F | Radiotherapy | 30 | Surgery | - | - | No/not referred | - | - | - | - | - |
| 6 | 83 | F | Radiotherapy | ** | Surgery | Chest | Topical and systemic corticoids | No/not referred | - | Not performed | Not performed | Not performed | Not performed |
| 6 | 86 | F | Radiotherapy | 15 | - | Lower limb | - | Yes | - | - | - | - | - |
| 6 | - | F | Radiotherapy | - | - | Breast | - | No/not referred | - | - | - | - | - |
| 6 | - | F | Radiotherapy | - | - | Breast | - | No/not referred | - | - | - | - | - |
| 6 | - | - | Radiotherapy | 2520 | - | Site of irradiation | - | No/not referred | - | - | - | - | - |
| 11 | 67 | M | - | - | Gliptins | Upper limb | Gliptin discontinuation | No/not referred | - | Subepidermal blister with eosinophils. | BP180 | IgA,IgG | IgG |
| 12 | 60 | M | - | - | Anti-PD1 | Genital | Topical, systemic and IL corticoids | No/not referred | - | Subepidermal blister with eosinophils. | BP180 | - | - |
| 13 | 77 | M | stoma | - | - | Abdomen (peristomal) | Topical corticoids | No/not referred | - | Subepidermal blister with eosinophils. | BP180 | IgG and C3 | - |
| 14 | 71 | F | UV-A | - | - | forehead | Topical and systemic corticoids | Yes | - | Subepidermal blister with eosinophils. | - | IgG and C3 | Negative |
| 15 | 92 | F | Photodynamic therapy | 90 | - | Lower limbs | Topical corticoids | No/not referred | - | Not performed | - | Not performed | IgG |
| 16 | 77 | F | Phototherapy | - | localized scleroderma + cutaneous lichen sclerosus | Upper limbs | Topical and systemic corticoids | No/not referred | - | Ulceration, rare eosinophils | BP180 | IgG and C3 | IgG, epidermal side |
| 17 | 61 | F | - | - | - | Abdomen | Topical antibiotics/antiseptics | No/not referred | - | Subepidermal blister with eosinophils. | BP180 | IgG,IgA, C3 (n-serrated) | IgA, epidermal side |
| 18 | 77 | F | Lymphedema | 17155 | recurring cellulitis | Lower limb | MMF | No/not referred | - | Subepidermal blister | Positive | IgG,IgA, C3 (n-serrated) | IgG, epidermal and dermal side |
| 19 | 26 | F | Chemical peeling | 20 | - | facial | Oral corticoids + topical tacrolimus | No/not referred | - | Subepidermal blister with eosinophils. | Negative | IgG and C3 | - |
| 20 | 44 | F | Subcorneal pustulosis | 2 | - | Lower parts of the legs | Topical and systemic corticoids | No/not referred | - | Subepidermal blister | - | Negative | Negative |
| 21 | 46 | F | UV exposure | - | - | nasal | Topical and systemic corticoids | No/not referred | - | Subepidermal blister with eosinophils. | - | IgG and C3 | IgG, epidermal side |
| 22 | 75 | F | - | - | - | lower limb | Topical corticoids | Yes | 5 | Subepidermal blister with eosinophils. | - | IgG and C3 | Compatible |
| 23 | 39 | F | surgery | 5475 | - | lower limbs | Topical corticoids | no/not referred | - | Subepidermal blister with eosinophils. | - | c3 | - |
| 24 | 87 | F | Stoma | 150 | Radiotherapy | Abdomen (peristomal) | Topical corticoids | no/not referred | - | Subepidermal blister with eosinophils. | - | IgG and C3 | Epidermal side |
| 25 | 7 | M | Surgery | - | - | penile | Corticoids | No/not referred | - | Subepidermal blister with rare eosinophils. | - | IgG and C3, epidermal side | - |
| 26 | 92 | F | - | - | - | lower limbs | Topical corticoids | No/not referred | - | Subepidermal blister with rare eosinophils. | - | IgG and C3 | Epidermal side |
| 26 | 80 | F | - | - | - | lower limb | Topical and systemic corticoids + tetracycline + niacinamide | No/not referred | - | Subepidermal blister with rare eosinophils. | - | IgG and C3 | Epidermal side |
| 26 | 79 | F | - | - | - | chest | Topical corticoids | No/not referred | - | Not performed | - | IgG and C3 | Negative |
| 27 | 72 | F | Non-filiated chronic itch | - | - | perineum and perianal area | Oral corticoids + topical tacrolimus | No/not referred | - | Subepidermal blister with eosinophils. | BP180 | IgG and C3 | C3, epidermal side |
| 28 | 77 | M | Stoma | 4380 | - | Abdomen (peristomal) | Chemotherapy (Lymphoma) | No/not referred | - | Subepidermal blister with eosinophils. | BP180, BP230 | IgG and C3 | Epidermal side |
| 29 | 49 | F | Thermal burns | 35 | - | Lower limb | Oral corticoids | Yes | 16 | Subepidermal blister with eosinophils. | - | IgG | - |
| 30 | 82 | F | - | - | Anti-PD1 | Lower limbs | Topical corticoids | Yes | - | Subepidermal blister with eosinophils. | BP180 | IgG, C3 | Epidermal side |
| 31 | 71 | M | Bacterial + fungal infection | - | mechanical trauma (socks) | lower limbs | topical and systemic corticoids | No/not referred | - | Subepidermal blister with eosinophils. | BP230 | IgG, C3 | IgG, C3 |
| 32 | 79 | F | Surgery | - | - | Abdomen | - | No/not referred | - | Positive | - | Compatible (IF studies) | Compatible (IF studies) |
| 33 | 76 | M | Hemodialysis fistulas | - | - | Upper limbs | Oral corticoids | No/not referred | - | Subepidermal blister with eosinophils. | Negative | IgG,IgM,C3 | - |
| 33 | 52 | F | Hemodialysis fistulas | - | - | Upper limb | Oral corticoids | No/not referred | - | Positive | Negative | IgG,IgM,C4 | - |
| 34 | 71 | M | Surgery | - | - | Lower limb (Stump) | Topical and systemic corticoids | Yes | 5 | Subepidermal blister with eosinophils. | - | IgG | Compatible |
| 34 | 91 | F | Bone fracture | 7300 | - | Upper limb | Topical corticoids + ACTH | No/not referred | - | Subepidermal blister with eosinophils. | - | IgG, C3 | Compatible |
| 35 | 83 | M | - | - | - | foot | - | No/not referred | - | Subepidermal blister with eosinophils. | BP230 | IgG, C3 | Positive |
| 36 | 65 | F | No | - | - | lower limbs | Topical corticoids | No/not referred | - | Subepidermal blister with eosinophils. | BP230 | IgG,IgE,C3 | IgG. C3 on epidermal side |
| 37 | 66 | M | - | - | - | lower limbs | Topical corticoids + tetracycline | No/not referred | - | Subepidermal blister with eosinophils. | - | IgG, C3 | Negative |
| 37 | 48 | M | - | - | a seizure disorder | lower limb | Topical and systemic corticoids + tetracycline | No/not referred | - | Subepidermal blister with eosinophils. | - | IgG, C3 | - |
| 38 | 77 | F | - | - | captopril | hands | Topical and systemic corticoids | yes | - | Blister with eosinophils. | - | - | - |
| 39 | 77 | M | Generalized pustular psoriasis | - | - | lower limb | MTX + topical corticoids | No/not referred | - | Subepidermal blister with eosinophils. | - | IgG, C3 | - |
| 40 | 46 | M | UV exposure | - | - | lower limb | Topical corticoids | No/not referred | - | Subepidermal blister with eosinophils. | - | IgM, C3 | IgG |
| 40 | 92 | M | - | - | - | lower limbs | topical triamcinolone | No/not referred | - | Subepidermal blister with eosinophils. | - | IgG, IgA, IgE, IgM, C3 | IgG |
| 40 | 68 | F | - | - | - | lower limb | - | No/not referred | - | - | BP230 | IgG, C3 | IgG |
| 41 | 59 | F | - | - | - | lower limbs | topical corticoids | No/not referred | - | - | - | IgG, C3 | IgG, epidermal side |
| 42 | 71 | F | Stoma | 1460 | - | Abdomen (peristomal) | tetracycline | No/not referred | - | Subepidermal blister with eosinophils. | - | C3 | - |
| 43 | 76 | - | - | - | - | lower limbs | topical corticoids | No/not referred | - | Dermal edema with eosinophils. | - | fibrinogen | IgG |
| 43 | 93 | - | - | - | - | lower limb | topical corticoids | No/not referred | - | Subepidermal blister with eosinophils. | BP230 | Negative | Compatible |
| 44 | 76 | F | - | - | - | lower limbs | topical corticoids | No/not referred | - | Dermal edema with eosinophils. | - | Negative | Compatible |
| 44 | 93 | F | - | - | - | lower limb | topical corticoids | No/not referred | - | Subepidermal blister with eosinophils. | - | Negative | Compatible |
| 44 | 84 | F | - | - | - | lower limbs | topical corticoids | No/not referred | - | Subepidermal blister | - | IgG, C3 | Compatible |
| 45 | 63 | M | Epidural injection | Post-surgical | Radiotherapy, surgery | back (injection site) | topical corticoids | yes | 9 | Subepidermal blister with eosinophils. | - | IgG, C3 | - |
| 46 | 79 | F | Herpes zoster | 30 | - | chest + right upper limb | oral corticoids | No/not referred | - | Subepidermal blister with eosinophils. | - | IgG, C3 | IgG, C3, epidermal side |
| 47 | 56 | F | - | - | - | upper limb | - | No/not referred | - | - | BP230 | Compatible | Compatible |
| 47 | 78 | M | - | - | - | chest | - | No/not referred | - | - | Negative | Compatible | Negative |
| 47 | 86 | M | - | - | - | perianal area | - | No/not referred | - | - | BP180, BP230 | Compatible | Compatible |
| 47 | 86 | F | - | - | - | lower limb | - | No/not referred | - | - | BP230 | Compatible | Compatible |
| 47 | 79 | F | - | - | - | lower limbs | - | No/not referred | - | - | BP230 | Compatible | Compatible |
| 47 | 75 | F | - | - | - | lower limbs | - | No/not referred | - | - | BP230 | Compatible | Compatible |
| 47 | 76 | F | - | - | - | lower limbs | - | No/not referred | - | - | BP230 | Compatible | Compatible |
| 47 | 95 | F | - | - | - | lower limbs | - | No/not referred | - | - | BP230 | Compatible | Compatible |
| 24 | 79 | M | - | - | - | lower limb | - | No/not referred | - | - | - | IgG, C3 | - |
| 24 | 72 | M | - | - | - | lower limb | - | No/not referred | - | - | - | C3 | - |
| 24 | 68 | M | - | - | - | lower limb | - | No/not referred | - | - | - | IgG, C3 | - |
| 24 | 83 | M | - | - | - | upper limbs | - | No/not referred | - | - | - | IgG, C3 | - |
| 24 | 84 | F | - | - | - | axilla | - | No/not referred | - | - | - | IgG, C3 | - |
| 24 | 90 | F | - | - | - | perineum | - | No/not referred | - | - | - | IgG, C3 | - |
| 24 | 65 | F | Injury | - | - | lower limb | Topical, systemic and IL corticoids | No/not referred | - | Subepidermal blister with eosinophils. | - | IgG, fibrin | Compatible |
| 24 | 50 | M | - | - | - | lower limbs | - | No/not referred | - | - | - | IgG, C3 | Not performed |
| 24 | 52 | F | - | - | - | upper limbs | - | No/not referred | - | - | - | IgG, C3 | Not performed |
| 24 | 69 | F | Injury | - | - | lower limb | - | No/not referred | - | - | - | IgG, C3, fibrin | Compatible |
| 24 | 73 | F | - | - | - | lower limbs | Corticoids | No/not referred | - | - | - | IgG, C3 | Negative |

* BP was diagnosed by DIF after generalization.; **LBP onset took place during Radiotherapy.

**Acronyms**: PS: Present study; M: Male; F: Female; DIF: Direct immunofluorescence, IIF: Indirect immunofluorescence; PEG: Percutaneous Endoscopic Gastrostomy; IL: Intralesional; UV: Ultraviolet; ACTH: Adrenocorticotropic hormone; MMF: Mycophenolate mofetil; MTX: Methotrexate; PD1: Programmed cell death protein 1.

**References**

1. Ständer S, Kasperkiewicz M, Thaçi D, Schmidt E, Zillikens D, Vorobyev A, et al. Prevalence and presumptive triggers of localized bullous pemphigoid. J Dermatol (2021) 48(8):1257-1261. doi: 10.1111/1346-8138.15912.

2. Mounsey SJ, Heelan K, Hughes S, Fawcett H, Bunker CB. Localized genital bullous pemphigoid. Clin Exp Dermatol (2018) 43(7):810-812. doi: 10.1111/ced.13612.

3. Truss A, Papalexandris S, Gardner S, Harvey R. Localised bullous pemphigoid overlying knee arthroplasty: a diagnostic challenge. BMJ Case Rep (2019) 12(4):e227440. doi: 10.1136/bcr-2018-227440.

4. Torchia D, Caproni M, Ketabchi S, Antiga E, Fabbri P. Bullous pemphigoid initially localized around a urostomy. Int J Dermatol. 2006 Nov;45(11):1387-9. doi: 10.1111/j.1365-4632.2006.03118.x.

5. Hafejee A, Coulson IH. Localized bullous pemphigoid 20 years after split skin grafting. Clin Exp Dermatol. (2005) 30(2):187-8. doi: 10.1111/j.1365-2230.2004.01689.x.

6. Nguyen T, Kwan JM, Ahmed AR. Relationship between radiation therapy and bullous pemphigoid. Dermatology (2014) 229(2):88-96. doi: 10.1159/000362208.

7. Seishima M, Izumi T, Kitajima Y. Antibody to bullous pemphigoid antigen 1 binds to the antigen at perilesional but not uninvolved skin, in localized bullous pemphigoid. Eur J Dermatol (1999) 9(1):39-42.

8. Ohata C, Shirabe H, Takagi K, Kawatsu T, Hashimoto T. Localized bullous pemphigoid after radiation therapy: two cases. Acta Derm Venereol (1997) 77(2):157. doi: 10.2340/0001555577157.

9. Knoell KA, Patterson JW, Gampper TJ, Hendrix JD Jr. Localized bullous pemphigoid following radiotherapy for breast carcinoma. Arch Dermatol (1998) 134(4):514-5. doi: 10.1001/archderm.134.4.514.

10. Jappe U, Bonnekoh B, Gollnick H. Guess what! Initially localized bullous pemphigoid at the irradiation site of breast carcinoma. Eur J Dermatol (1999) 9(2):139-41.

11. Oya K, Fujii M, Taguchi S, Nishie W, Izumi K, Shimizu H. Localized bullous pemphigoid associated with dipeptidyl peptidase-4 inhibitor treatment. Eur J Dermatol (2018) 28(2):250-251. doi: 10.1684/ejd.2018.3230.

12. Alvarado SM, Weston G, Murphy MJ, Stewart CL. Nivolumab-induced localized genital bullous pemphigoid in a 60-year-old male. J Cutan Pathol (2022) 49(5):468-471. doi: 10.1111/cup.14183.

13. Batalla A, Peón G, De la Torre C. Localized bullous pemphigoid at urostomy site. Indian J Dermatol Venereol Leprol (2011) 77(5):625. doi: 10.4103/0378-6323.84067.

14. Pfau A, Hohenleutner U, Hohenleutner S, Eckert F, Landthaler M. UV-A-provoked localized bullous pemphigoid. Acta Derm Venereol (1994) 74(4):314-6. doi: 10.2340/0001555574314316.

15. Rakvit P, Kerr AC, Ibbotson SH. Localized bullous pemphigoid induced by photodynamic therapy. Photodermatol Photoimmunol Photomed (2011) 27(5):251-3. doi: 10.1111/j.1600-0781.2011.00609.x.

16. Maglie R, Baffa ME, Montefusco F, Pipitò C, Senatore S, Capassoni M, et al. Case Report: Bullous Pemphigoid Associated With Morphea and Lichen Sclerosus: Coincidental Diseases or Pathogenetic Association? Front Immunol (2022) 13:887279. doi: 10.3389/fimmu.2022.887279.

17. Schmidt E, Benoit S, Bröcker EB. Bullous pemphigoid with localized umbilical involvement. Acta Derm Venereol (2009) 89(4):419-20. doi: 10.2340/00015555-0644.

18. Perez A, Clements SE, Benton E, Robson A, Bhogal B, Stefanato CM, et al. Localized bullous pemphigoid in a patient with primary lymphoedema tarda. Clin Exp Dermatol (2009) 34(8):e931-3. doi: 10.1111/j.1365-2230.2009.03722.x.

19. Gu A, Zhang L, Ma F, Kong X. Induction of localized bullous pemphigoid on a young woman following a chemical peel. Indian J Dermatol Venereol Leprol (2021) 87(5):706-708. doi: 10.25259/IJDVL_1116_20.

20. Bernstein JE, Medenica M, Soltani K. Coexistence of localized bullous pemphigoid, morphea, and subcorneal pustulosis. Arch Dermatol (1981) 117(11):725-7.

21. Lee CW, Ro YS. Sun-induced localized bullous pemphigoid. Br J Dermatol (1992) 126(1):91-2. doi: 10.1111/j.1365-2133.1992.tb08414.x.

22. Borradori L, Prost C, Wolkenstein P, Bernard P, Baccard M, Morel P. Localized pretibial pemphigoid and pemphigoid nodularis. J Am Acad Dermatol (1992) 27(5 Pt 2):863-7. doi: 10.1016/0190-9622(92)70268-k.

23. Sen BB, Ekiz Ö, Rifaioglu EN, Sen T, Atik E, Dogramaci AÇ. Localized bullous pemphigoid occurring on surgical scars. Indian J Dermatol Venereol Leprol (2013) 79(4):554. doi: 10.4103/0378-6323.113111.

24. Salomon RJ, Briggaman RA, Wernikoff SY, Kayne AL. Localized bullous pemphigoid. A mimic of acute contact dermatitis. Arch Dermatol (1987) 123(3):389-92. doi: 10.1001/archderm.123.3.389.

25. Mirza M, Zamilpa I, Wilson JM. Localized penile bullous pemphigoid of childhood. J Pediatr Urol (2008) 4(5):395-7. doi: 10.1016/j.jpurol.2008.02.008.

26. Tran JT, Mutasim DF. Localized bullous pemphigoid: a commonly delayed diagnosis. Int J Dermatol (2005) Nov;44(11):942-5. doi: 10.1111/j.1365-4632.2004.02288.x.

27. Patsatsi A, Lazaridou E, Papagaryfallou I, Sotiriadis D. Bullous pemphigoid of the perineum and perianal area: a rare localized form in adults. Acta Derm Venereol (2008) 88(4):401. doi: 10.2340/00015555-0445.

28. Egan CA, Florell SR, Zone JJ. Localized bullous pemphigoid in a patient with B-cell lymphoma. South Med J. (1999) 92(12):1220-2. doi: 10.1097/00007611-199912000-00019.

29. Wagner GH, Ive FA, Paraskevopoulos S. Bullous pemphigoid and burns: the unveiling of the attachment plaque? Australas J Dermatol (1995) 36(1):17-20. doi: 10.1111/j.1440-0960.1995.tb00918.x.

30. Amber KT, Valdebran M, Lu Y, De Feraudy S, Linden KG. Localized pretibial bullous pemphigoid arising in a patient on pembrolizumab for metastatic melanoma. J Dtsch Dermatol Ges (2018) 16(2):196-198. doi: 10.1111/ddg.13411.

31. Kohroh K, Suga Y, Mizuno Y, Ishii N, Hashimoto T, Ikeda S. Case of localized bullous pemphigoid with unique clinical manifestations in the lower legs. J Dermatol (2007) 34(7):482-5. doi: 10.1111/j.1346-8138.2007.00315.x.

32. Massa MC, Freeark RJ, Kang JS. Localized bullous pemphigoid occurring in a surgical wound. Dermatol Nurs (1996) 8(2):101-3.

33. Pardo J, Rodrguez-Serna M, Mercader P, Fortea JM. Localized bullous pemphigoid overlying a fistula for hemodialysis. J Am Acad Dermatol (2004) 51(2 Suppl):S131-2. doi: 10.1016/j.jaad.2004.03.024.

34. Macfarlane AW, Verbov JL. Trauma-induced bullous pemphigoid. Clin Exp Dermatol (1989) 14(3):245-9. doi: 10.1111/j.1365-2230.1989.tb00944.x.

35. Domloge-Hultsch N, Utecht L, James W, Yancey KB. Autoantibodies from patients with localized and generalized bullous pemphigoid immunoprecipitate the same 230-kd keratinocyte antigen. Arch Dermatol (1990) 126(10):1337-41.

36. Soh H, Hosokawa H, Miyauchi H, Izumi H, Asada Y. Localized pemphigoid shares the same target antigen as bullous pemphigoid. Br J Dermatol (1991) 125(1):73-5. doi: 10.1111/j.1365-2133.1991.tb06045.x.

37. Thornfeldt CR, Menkes AW. Bullous pemphigoid controlled by tetracycline. J Am Acad Dermatol (1987) 16(2 Pt 1):305-10. doi: 10.1016/s0190-9622(87)70040-1.

38. Mallet L, Cooper JW, Thomas J. Bullous pemphigoid associated with captopril. DICP (1989) 23(1):63. doi: 10.1177/106002808902300115.

39. Iskandarli M, Gerceker Turk B, Yaman B, Ozturk G. Pemphigoid Diseases as a Sign of Active Psoriasis: A Case Report and Brief Review. Dermatology (2015) 231(4):319-21. doi: 10.1159/000435912.

40. Provost TT, Maize JC, Ahmed AR, Strauss JS, Dobson RL. Unusual subepidermal bullous diseases with immunologic features of bullous pemphigoid. Arch Dermatol (1979) 115(2):156-60.

41. Seishima M, Izumi T, Kitajima Y. Antibody to bullous pemphigoid antigen 1 binds to the antigen at perilesional but not uninvolved skin, in localized bullous pemphigoid. Eur J Dermatol (1999) 9(1):39-42.

42. Vande Maele DM, Reilly JC. Bullous pemphigoid at colostomy site: report of a case. Dis Colon Rectum (1997) 40(3):370-1. doi: 10.1007/BF02050431.

43. Muramatsu T, Iida T, Shirai T. Antibasement membrane zone antibodies in localized pretibial pemphigoid. Int J Dermatol (1991) 30(6):422-4. doi: 10.1111/j.1365-4362.1991.tb03898.x.

44. Muramatsu T, Iida T, Shirai T. Pemphigoid and pemphigus foliaceus successfully treated with topical corticosteroids. J Dermatol (1996) 23(10):683-8. doi: 10.1111/j.1346-8138.1996.tb02681.x.

45. Parslew R, Verbov JL. Bullous pemphigoid at sites of trauma. Br J Dermatol (1997) 137(5):825-6. doi: 10.1111/j.1365-2133.1997.tb01130.x.

46. Gurel MS, Savas S, Bilgin F, Erdil D, Leblebici C, Sarikaya E. Zosteriform pemphigoid after zoster: Wolf's isotopic response. Int Wound J (2016) 13(1):141-2. doi: 10.1111/iwj.12423.

47. Kawahara Y, Matsumura K, Hashimoto T, Nishikawa T. Immunoblot analysis of autoantigens in localized pemphigoid and pemphigoid nodularis. Acta Derm Venereol. 1997 May;77(3):187-90. doi: 10.2340/0001555577187190.
